# A novel R-package graphic user interface for the analysis of metabonomic profiles

**DOI:** 10.1186/1471-2105-10-363

**Published:** 2009-10-29

**Authors:** Jose L Izquierdo-García, Ignacio Rodríguez, Angelos Kyriazis, Palmira Villa, Pilar Barreiro, Manuel Desco, Jesús Ruiz-Cabello

**Affiliations:** 1 Instituto de Estudios Biofuncionales, UCM, Madrid, Spain; 2 CIBER de enfermedades respiratorias, Madrid, Spain; 3 Departamento de Ingeniería Rural, ETSI Agrónomos, Universidad Politécnica de Madrid, Madrid, Spain; 4 Unidad de Medicina y Cirugía Experimental, Hospital General Universitario Gregorio Marañón, Madrid, Spain

## Abstract

**Background:**

Analysis of the plethora of metabolites found in the NMR spectra of biological fluids or tissues requires data complexity to be simplified. We present a graphical user interface (GUI) for NMR-based metabonomic analysis. The "Metabonomic Package" has been developed for metabonomics research as open-source software and uses the R statistical libraries.

**Results:**

The package offers the following options:

Raw 1-dimensional spectra processing: phase, baseline correction and normalization.

Importing processed spectra.

Including/excluding spectral ranges, optional binning and bucketing, detection and alignment of peaks.

Sorting of metabolites based on their ability to discriminate, metabolite selection, and outlier identification.

Multivariate unsupervised analysis: principal components analysis (PCA).

Multivariate supervised analysis: partial least squares (PLS), linear discriminant analysis (LDA), k-nearest neighbor classification.

Neural networks.

Visualization and overlapping of spectra.

Plot values of the chemical shift position for different samples.

Furthermore, the "Metabonomic" GUI includes a console to enable other kinds of analyses and to take advantage of all R statistical tools.

**Conclusion:**

We made complex multivariate analysis user-friendly for both experienced and novice users, which could help to expand the use of NMR-based metabonomics.

## Background

Decoding the genome (genomics) is not sufficient to explain the cause of many diseases. Therefore, the study of differences in gene expression between subjects (transcriptomics), the analysis of protein synthesis (proteomics), and the study of metabolic regulation (metabolomics) have been intensified in recent years [1].

Analysis of the plethora of metabolites found in the NMR spectra of biological fluids or tissues requires data complexity to be reduced [2,3]. The field of metabonomics is evolving in parallel to the application of multivariate statistical methods with this purpose.

However, multivariate analysis is not easy for novice users. Several commercial programs can help such users apply multivariate methods, although none include the full range of routines, from data pre- and post-processing to the final statistical results. Recently, an open-source platform (Automics) [4] based on Visual C++ has been developed to carry out a full NMR-based metabonomic analysis. Automics includes the most common 1D NMR spectral processing functions and nine statistical methods: feature selection (Fisher's criterion), data reduction (PCA, LDA, uncorrelated LDA), unsupervised clustering (K-Means) and supervised regression and classification methods (PLS/PLS-DA, KNN, Soft Independent Modellingof Class Analogy [SIMCA], Support Vector Machines [SVM]).

We present a new software package based on **the open-source R framework **[5] with a graphical user interface (GUI) that helps the user understand and run such methods for the analysis of NMR-based metabonomic data. Our package is called "Metabonomic" and it makes use of different R libraries to build a statistics toolbox. Moreover, the R framework open-source architecture allows newly proposed algorithms or methods for spectral processing and data analysis to be implemented and included much more easily and freely accessed by the public. The "Metabonomic" GUI includes unsupervised multivariate analysis techniques (eg, principal components analysis [PCA]), supervised multivariate analysis (eg, partial least squares [PLS] analysis, linear discriminant analysis (LDA), and k-nearest neighbor classification). It can also be used to define different types of neural networks. In our study, we test some of these multivariate methods using internal cross-validation and external validation.

This "Metabonomic" package also enables pre-processing of raw NMR spectra. Pre-processing transforms the data in such a way that subsequent analysis and modelling are easier, more robust, and more accurate. In the analysis of NMR spectra, pre-processing methods usually attempt to reduce variance and any other possible source of bias such as phase correction, peak shifting or misalignment, and baseline correction. Although the "Metabonomic" package has been developed for the analysis of NMR spectra, this software can also be used for the pre-processing of mass spectrometry-based profiles or other 1-dimensional spectra. The analysis of 2-dimensional NMR spectra will be available in the next software update.

## Implementation

### Program Description

The "Metabonomic" GUI was designed using the R-Tcl/Tk interface [6,7], which enables us to use the TK toolkit and replace Tcl code with R function calls to facilitate interaction with the R functions and a comprehensive metabonomic analysis. The software offers several graphic outputs, through plots created using a combination of different Tcl/Tk interfaces. The program is based on R version 2.8.0 [5] under the Windows operating system.

The "Metabonomic" GUI, requires packages [Table 1] to be downloaded and installed in the R console. The PROcess package can be found at the Bioconductor Project Site [8]. Once the required packages are ready, the "Metabonomic" package is loaded using the Package installer or writing ">require (Metabonomic)" if the package is already in the computer.

**Table 1:** Packages required to execute the Metabonomic GUI

	**R packages**
**Graphical Integration**	tcltk, tcltk2, tkrplot, scatterplot3d

**Pre-processing**	PROcess, caMassClass, FTICRMS, clusterSim, waved

**Multivariate**	hddplot, MASS, gpls, pls, class, robustbase, relimp, Icens

**Neural Networks**	nnet, AMORE, neural

The program is started by writing "> Metabonomic()" in the R console to open the main user interface. The GUI has an input console, which can be used to launch any R application, and two different output consoles, where warnings and output messages are displayed. It also has a button line, with the following buttons: (a) undo, (b) redo, (c) current data display, (d) launch the commands written in the input console, (e) erase the input console, (f) stop any running process, and (g) shut down the GUI and return to the R console.

Finally, the GUI has a main menu with different tabs: File, Script, Edit, Pre-processing, Metabonomic Analysis, and Spectrum. The Script tab provides access to the following functions: (a) "Load a Script," which opens a script into the input console, (b) "Save Script," which saves the commands written in the input console as an R script file, and (c) "Launch the Script," which runs the commands written in the input console. Other functions are described in detail in the following sections.

### Data Importing

The NMR processed spectra for metabonomic analysis are loaded as a text file by selecting the "file/Load Data file" tab. The text file, with no header, shows the chemical shift (in ppm) in the first column, and the intensities of the different spectra are in the following columns. After importing the spectra text file, the GUI asks for an "info" file. This file contains all the sample information, which has been previously written by the user as a text file, where the first column holds the names of the samples and the different characteristics are in the following columns separated by tabs [Table 2]. A header with the caption of each column is also required.

**Table 2:** Example of an info file

**Name**	**Category**	**Exposure**
RF03	Tobacco	Chronic
RF08	Tobacco	Chronic
RF10	Tobacco	Chronic
RF13	Tobacco	Chronic
RF16	Tobacco	Chronic
RF17	Tobacco	Chronic
RF20	Tobacco	Chronic
RF27	Tobacco	Chronic
RF30	Tobacco	Chronic
RF31	Tobacco	Chronic
RF32	Tobacco	Chronic
RF33	Tobacco	Chronic
RF47	Tobacco	Chronic
RF48	Tobacco	Chronic
RF49	Tobacco	Chronic
RF01	Control	Control
RF02	Control	Control
RF04	Control	Control
RF07	Control	Control
RF12	Control	Control
RF21	Control	Control
RF28	Control	Control
RF43	Control	Control
RF44	Control	Control
RF45	Control	Control
RF52	Control	Control
RF53	Control	Control
RF54	Control	Control

Alternatively, the data can be loaded directly from the Bruker spectroscopy format by an independent package that can be executed by selecting the "file/Import Bruker file" tab. The user has to select the raw data (FID file in the Bruker data directory). This application displays the spectrum reference and manages basic operations such as setting the chemical shift of a certain compound (trimethylsilylpropionic acid or dimethylsilapentane sulfonic acid) to 0 ppm and zero order and first order phase corrections[9]. When the first set of data is loaded, the GUI asks for a new array. When all the spectra are imported, the GUI asks for the "info" file. Applications to load other commercial data formats will be added soon.

The GUI also allows processed data to be exported as a text file.

#### Category Selection

This application selects the information that will be used in the supervised analysis. First, the GUI asks which characteristic (different columns of the info file) will be used to classify the samples. The user then chooses the different types of samples that will be used in the multivariate analysis. To date, the program only allows the selection of four different sample types. The "Category Selection" application is launched by selecting the "file/Category Selection" tab.

### Data Pre-Processing

Data must be pre-processed carefully, since any inaccuracy introduced at this stage can cause significant errors in the multivariate analysis. Thus, the GUI offers several guided corrections, as explained below. If any special correction or data processing is necessary, it can be easily programmed in the input console.

#### Region Exclusions

The first step of data pre-processing usually involves the exclusion of spectral regions [10], which either contain non-reproducible information or do not contain information about metabolites. On the one hand, the spectral width to acquire NMR data is usually wider than necessary to digitize all chemical shifts associated with endogenous metabolites. Thus, downfield and upfield spectral areas without any endogenous metabolites are initially excluded. On the other hand, spectral regions highly depending on the experimental parameters, such as the water and the reference regions are also deleted. As these regions are sensitive to spectral artifacts, such as inadequate phasing, exclusion is beneficial. Therefore, the spectrum outside the 0.2-10-ppm window is usually excluded. By selecting the "file/Manual Cut" tab, a graphical application to select the area of interest in the spectrum and to delete the water resonance region is launched.

#### Baseline Correction

Baseline correction is an essential step to obtain high quality NMR spectra in some cases [11,12]. Rolling baselines can make it difficult to identify peaks and can introduce significant errors into any quantitative measurements. In order to avoid errors, the GUI incorporates an application to reduce this influence in batch mode. Baseline correction is performed using the "bslnoff" function, which is based on the LOESS method [13] from the PROcess library [8]. This graphical application (Pre-processing/Baseline) allows the bandwidth to be controlled so that it can be passed to the LOESS function until the adjustment is correct. Graphs with the raw spectrum, estimated baseline, and baseline-subtracted spectrum are plotted in the R console.

**Figure 1:**
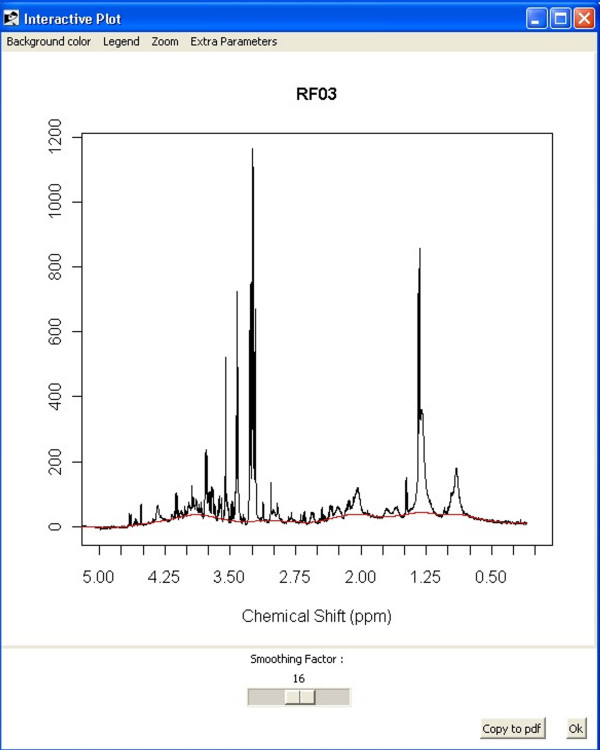
**Baseline (FTICRMS) display**. Baseline correction of a proton-NMR spectrum using the Baseline (FTICRMS) display.

Another application, based on the FTICRMS package [14], is available for individual baseline correction. It computes an estimated baseline curve for a spectrum using the method of Rocke and Xi [15]. The most important parameter for obtaining a perfect baseline is the smoothing parameter, which is controlled by a slider widget. The algorithm uses extra parameters that have been optimized for NMR data sets, such as negativity penalty, maximum number of iterations, or a parameter for robust center and scale estimation. In any case, these parameters can be modified through the "Extra Parameters" tab. All changes are instantly displayed in the graphical device [Figure 1], thus allowing an interactive baseline adjustment.

#### Binning

The most common method of reducing the influence of shifting peaks is the so-called binning or bucketing method, which reduces spectrum resolution [16]. Thus, the spectra are integrated within small spectral regions, called "bins" or "buckets". Subsequent data analysis procedures applied to the binned spectra are not influenced by peak shifts, as long as these shifts remain within the borders of the corresponding bins. After launching the binning graphical applications (Pre-processing/Binning), the user can select the bin size. This process is executed by the "binning" function from the PROcess library [8].

#### Peak detection and alignment

Peak alignment is an alternative to binning the spectrum to account for peak shifts [10,17,18]. A peak detection graphical application (Pre-processing/Peak Detection) has been developed to control the "msc.peaks.find" function from the caMassClass library [19]. The graphical application adjusts the signal-to-noise ratio and the threshold criterion in the peak's detection process and returns a data frame with the positions and intensities of the detected peaks. These are aligned by a peak alignment graphical application (Pre-processing/Peak Alignment). This application guides the user in the use of the "msc.peaks.align" function from the caMassClass library [19].

#### Normalization

A crucial step in pre-processing of spectrum data in metabonomic studies is the so-called normalization step [10]. This step tries to account for possible variations in sample concentrations. Normalization may also be necessary for technical reasons. If spectra are recorded using a different number of scans or different devices, the absolute values of the spectra vary, and rendering a joint analysis of spectra without prior normalization is impossible. The normalization graphical application (Preprocessing/Normalization) makes it possible to choose between several types of normalization steps using functions from the clusterSim library [20].

### Principal Components Analysis

Principal components analysis (PCA) is one of the most common exploratory steps in multivariate analysis [21-23], and its most important use is to represent multivariate data in a low-dimensional space. The first principal component is the maximum variation direction in the cluster of points. The second principal component is the second largest variation, and so on.

**Figure 2:**
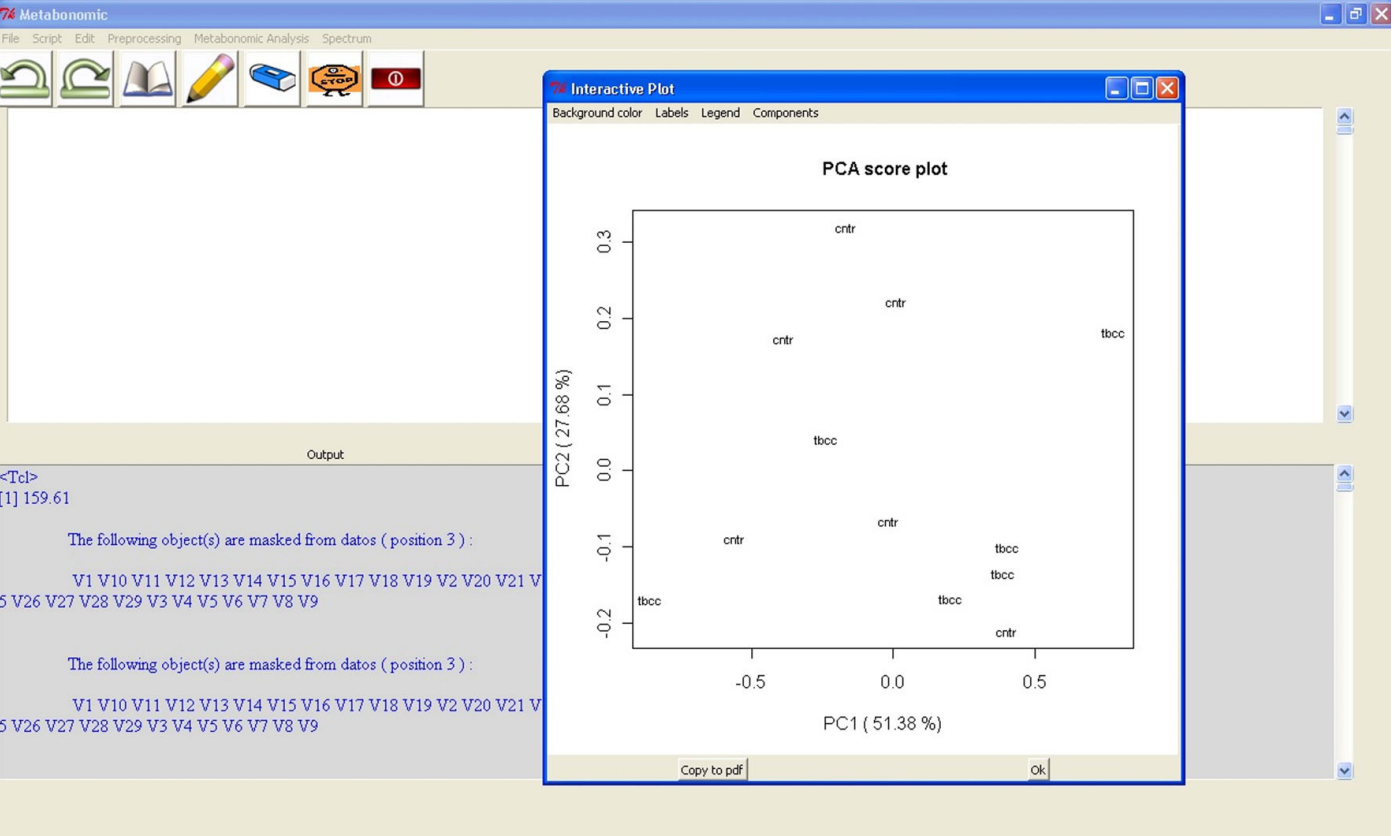
**Metabonomic GUI used for PCA**. First and second principal component score plot of two class samples (control and tobacco).

The GUI incorporates a PCA graphical application (Metabonomic Analysis/PCA) to guide users in PCA by allowing the selection of the algorithm parameters. In addition, interactive graphics have been developed to change items such as the component and graphical parameters in the score [Figure 2] and loading plots. The principal components algorithm used is based on the "prcomp" function from the stats library [24].

In addition, a graphical display for outlier identification has been developed using the "prcomp" function and the "robustbase" package [25] (pre-processing/outliers). It shows Mahalanobis distances based on robust and classic estimates of the location and the covariance matrix in different plots.

### Linear Discriminant Analysis

Linear discriminant analysis (LDA) is another common technique for the analysis of metabonomic data [21,26]. It is used to obtain linear discriminant functions, a linear combination of the original classes chosen to maximize the differences between them. For samples with only two classes, the discriminating function is a line, for three classes it is a plane, and for more than three classes a hyperplane. In the LDA graphical application (Metabonomic Analysis/LDA), the linear discriminant function is calculated by the "lda" function from the "MASS" package [27,28].

**Figure 3:**
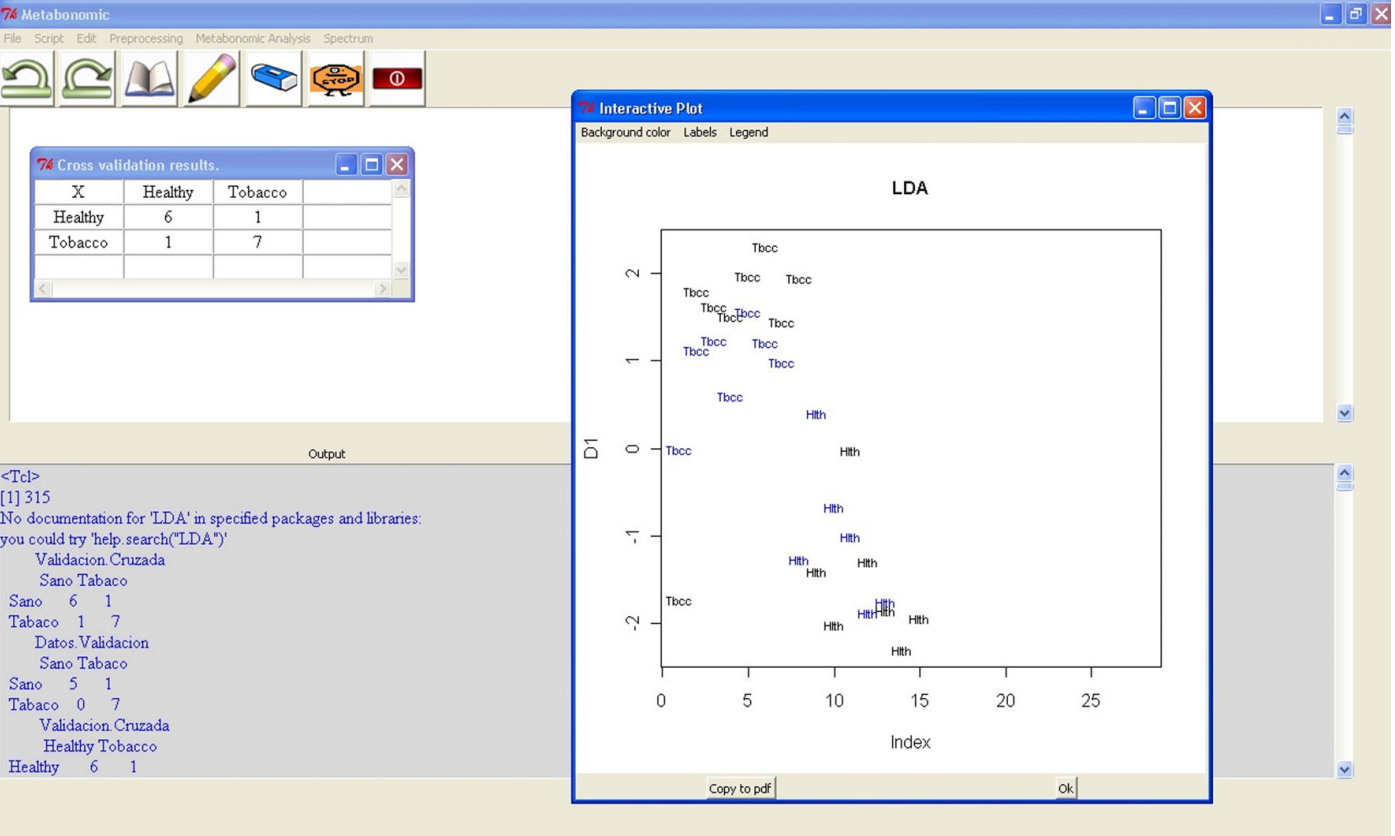
**Metabonomic GUI used for LDA**. LDA score plot of two class samples (control and tobacco) with the training model samples (black) and the testing model samples (blue). The cross-validation result is also returned.

The program guides the user through the tasks in the proper order. First, an LDA model is built with part of the samples; the remainder are used to perform a validation test. The user can choose the samples directly to make the model, or randomly select the number of samples from each class. Second, the user can select the algorithm to calculate the LDA from among the following: "moment" for standard estimators of the mean and variance, "mle" for a maximum likelihood estimation, or "t" for robust estimates based on a t distribution. Finally, the LDA graphical application returns the results of the validation test and different interactive graphs of the LDA model [Figure 3]. If the number of different classes is three or less, the interactive graph is a plane where the samples used to build the model and the validation samples are plotted. If the number of different classes is greater than three, the samples used to build the model and the validation samples are plotted in interactive cubes. In these interactive plots, the user can select the angle of rotation, the components shown, and other graphical parameters.

### Partial Least Squares Discriminant Analysis

Another common multivariate method [21,29,30] in metabonomic analysis is partial least squares discriminant analysis (PLS-DA), a supervised linear regression method whereby the multivariate variables corresponding to the observations (spectral descriptors) are associated with the class membership for each sample [31]. PLS-DA provides an easily understandable graphical approach to identifying the spectral regions of difference between the classes, and allows a statistical evaluation of whether the differences between classes are significant.

Two different PLS-DAs have been included in the "Metabonomic" GUI. The first PLS graphical application (Metabonomic Analysis/Partial Least Squares/PLS) was developed with a PLS algorithm based on the extension of the generalized partial least squares model proposed by Ding and Gentleman [32]. This algorithm is implemented using the "gpls" function from the "gpls" package [33], and it allows separation between no more than two classes of samples. The graphical application controls the manual or random selection of the samples to build the model, the selection of all the algorithm parameters such as the tolerance to the convergence, the number of iterations allowed, and the number of PLS components used. At the end, the results of the validation test are returned.

The second application (Metabonomic Analysis/Partial Least Squares/PLS with graphics) is performed using the "plsr" function from the "pls" package [34,35]. This PLS-DA is more complex, and the application guides the user through all the steps in the proper order. First, the user chooses between manual and random selection of the samples. Second, the user selects the PLS algorithm and the validation method. The four PLSR algorithms available are the kernel algorithm [36], the wide kernel algorithm [37], the SIMPLS algorithm [38], and the classic orthogonal scores algorithm [39].

Next, the application creates a PLS model with the maximum number of components and shows the explained variance and the R^2 ^graphics of the model. With this information, the user can select the optimum number of PLS components to build the model. In addition, the standard error of prediction (SEP) and the root mean standard error of prediction (RMSEP) are plotted in the R console.

**Figure 4:**
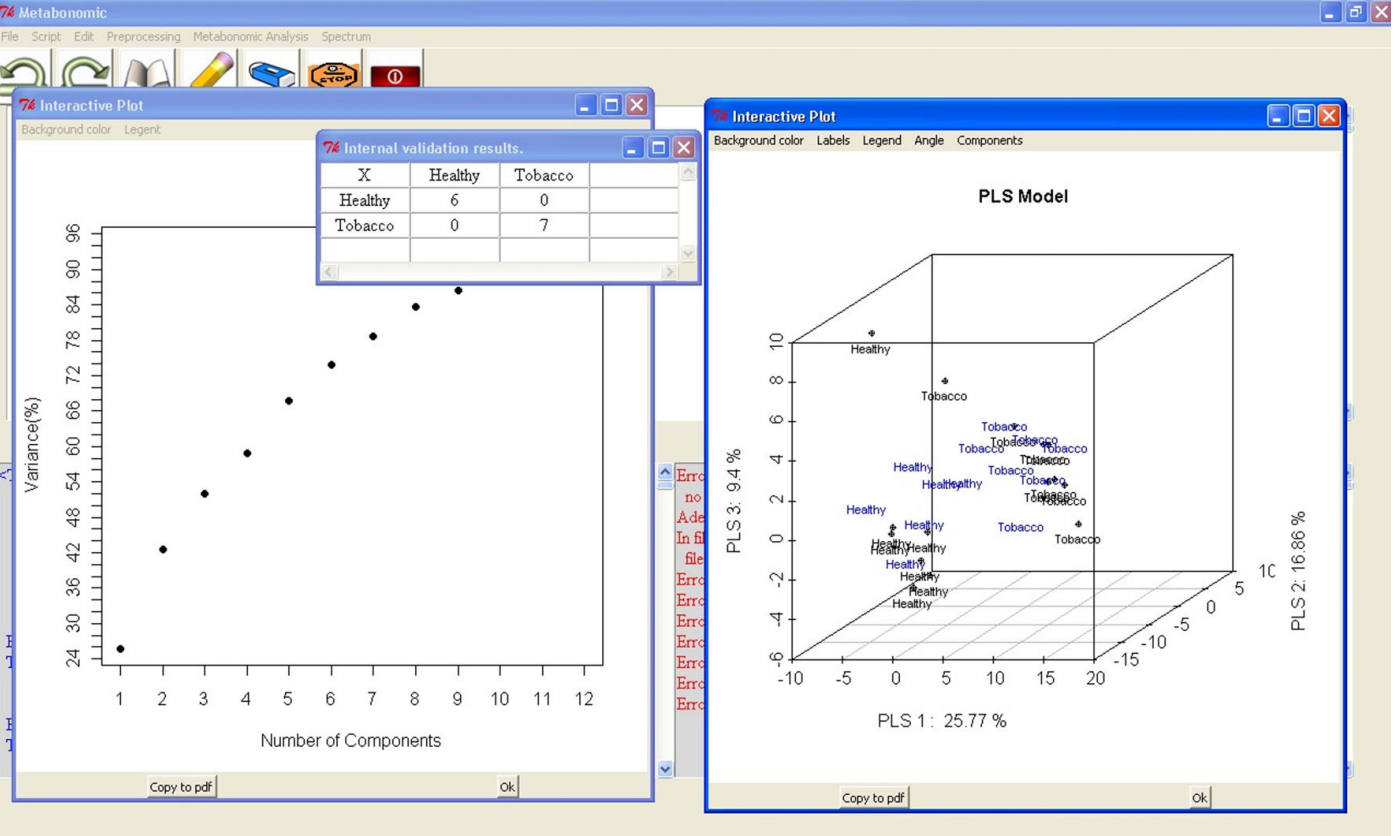
**Metabonomic GUI used for PLS-DA**. Interactive graph (right) with the first three PLS components score plot of two classes of samples (healthy and tobacco). The black samples are the samples used to build the model and the blue samples are the validation samples. In addition, the validation result and the explained variance are shown.

Finally, the PLS graphical application returns the results of the validation test and different interactive graphs of the PLS model [Figure 4].

### K-Nearest Neighbors Classification

The k-nearest neighbors (KNN) rule for classification [40] is the simplest of all supervised classification approaches. For the classification of an unknown object, its distance (usually the Euclidian distance) to all other objects is computed. The minimum distance is selected and the object is assigned to the corresponding class. The KNN graphical interface (Metabonomic Analysis/KNN) allows the user to choose between random or manual selection of the samples to build the model, number of neighbors, minimum vote for definite decision, and the use or not of all the neighbors. If the all the neighbors are used, all distances equal to the kth largest are included. If not, a random selection of distances equal to the kth is chosen to use exactly k neighbors. To finish, the interface returns the results of the validation test and the cross-validation test. The KNN graphical application uses the "knn" function from the class package [28].

### Neural Networks

Application of artificial neural networks (ANNs) for data processing is characterized by analogy with a biological neuron. An ANN consists of a layered network of nodes, each of which performs a simple operation on several inputs to produce a single output.

Two different applications to define ANNs have been included in the "Metabonomic" GUI. The first application (Metabonomic Analysis/Neural Network/Neural Network [Single hidden layer]) makes use of the "nnet" function from the "nnet" R package [28]. This graphical application allows the user to build a single-hidden-layer neural network, by selecting the number of units in the hidden layer, the initial random weight, and the weight decay. In addition, the user can choose between random or manual selection of the training samples.

The second application (Metabonomic Analysis/Neural Network/Neural Network [multiple hidden layers]) creates a feedforward artificial neural network according to the structure established by the "AMORE" package [41]. With this application, the user can select the number of layers and the number of neurons in each layer, while controlling several parameters. These include the learning rate at which every neuron is trained, the momentum for every neuron, the error criterion (least mean squares or least mean logarithm squares), the activation function of the hidden and the output layer (Purelin, Tansig, Sigmoid, or Hardlim), and the training method (Adaptive gradient descent or BATCH gradient descent, with or without momentum). With these parameters selected, the algorithm trains the network with the manually or randomly selected samples before testing it with the rest of the samples.

### Other Tools

In addition to the multivariate techniques, other useful graphical tools have been developed in the "Metabonomic" GUI to enable easy interpretation of complex data tables.

**Figure 5:**
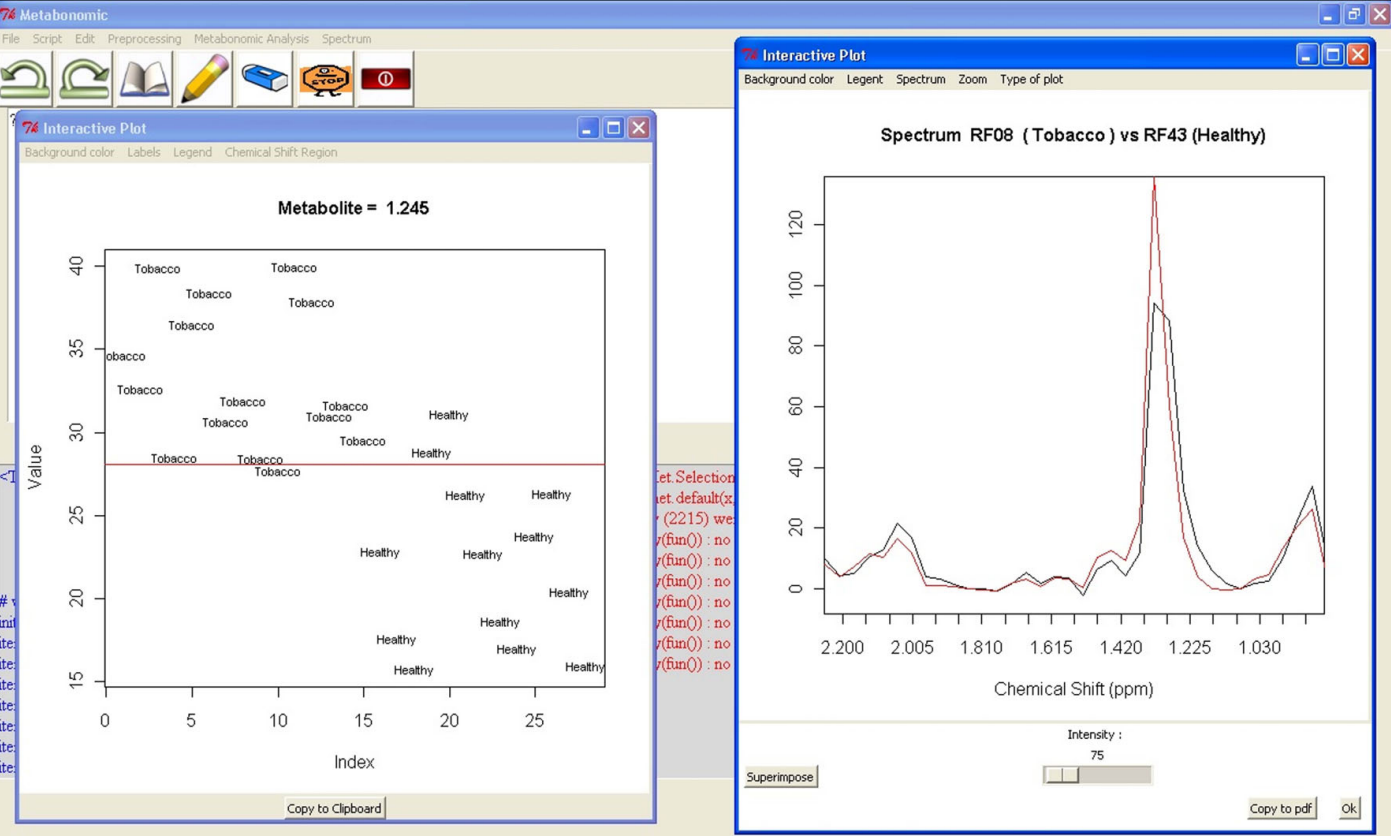
**Extra tools**. Metabonomic GUI tools to visualize and overlap the spectra (right) and to show the values of all samples in a given chemical position (left).

For example, a graphical display (Metabonomic/Chemical Shift Region Display) has been added to show the differences between the subgroups in a specific spectral region. The application plots the values and means of all samples in the specified chemical shift region [Figure 5].

Another graphical display (Spectrum/...) has been created to visualize and overlap the spectra. With these applications, the user can focus the interesting areas with a zoom tool, superimpose different spectra, increase or decrease the spectra intensity, and change other graphical parameters. Moreover, when the user clicks with the cross cursor in the spectrum, a new window pops up showing the chemical shift and the intensity of this selected resonance. This display can be launched for the original or for the current spectra [Figure 5].

## Results

An NMR analysis of lung tissue was used to test our package. This dataset (unpublished data) consisted of 28 AKR/J mice chronically exposed to tobacco smoke for 5 days/week (n = 15) over a 6-month period and a sham group (n = 12).

High-resolution magic angle spinning spectra were generated from intact lung tissue using a BRUKER AMX500 spectrometer 11.7 T, 500.13 MHz (256 scans collected for each sample, 16K data points).

First, the water peak and the spectrum area outside the 0.2-10-ppm window were removed. The baseline of each spectrum was corrected using the Baseline (FTICRMS) tool. In addition, the spectra were normalized by total area and integrated within 0.04-ppm buckets.

The pre-processed spectra underwent different multivariate analyses. The multivariate models were built with a number of random training samples (8 samples of each type). The remaining samples can be used to perform a validation test, derived from the probability of belonging to each group. The validation results are summarized in Table 3.

**Table 3:** Validation results for different multivariate methods incorporated in the GUI

**Multivariate Method**	**Tobacco predictive value**	**Healthy predictive value**	**Sensitivity**	**Specificity**
LDA	86%	100%	100%	83%

**PLS (Ding and Gentleman)**	100%	100%	100%	100%

PLS (Kernel)	100%	100%	100%	100%

**Single hidden layer neural network**	86%	100%	100%	83%

Feedforward neural network	86%	100%	100%	83%

## Conclusion

Pre-processing of raw NMR spectra and different multivariate analyses are standard procedures applied to interpret the complex metabonomic profile. The "Metabonomic" GUI presented in this paper offer an easy application of the principal pre-processing methods and the most commonly used multivariate statistical methods in metabonomic analysis. Various tools have been developed or adapted to make statistical analysis easier for the inexperienced user. The more experienced user always maintains complete control of the statistical tools. Special correction or data processing can be carried out using the input console.

The main advantage of the "Metabonomic" GUI is its modular design, which makes it easy to upgrade. Furthermore, new analysis methods can be included in the metabonomic field using the large R free software library.

## Availability and requirements

• Project name: Metabonomic R package.

• Project home page: 

• Operating system: MS Windows.

• Programming language: R. The package runs on MS Windows using an installed version of R.

• Other requirements: The required PROcess package is available in the Bioconductor website .

• Licence: GPL version 2 or newer.

## List of abbreviations

ANN: artificial neural network; GUI: graphical user interface; KNN: k-nearest neighbors; LDA: linear discriminant analysis; PCA: principal components analysis; PLS: partial least squares; PLS-DA: partial least squares discriminant analysis; NMR: nuclear magnetic resonance; GUI: graphical user interface.

## Authors' contributions

JLIG carried out the programming and software design and drafted the manuscript. PV, IR, AK, PB, and MD provided domain knowledge and helped to draft the manuscript. JRC conceived the study, participated in its design and coordination, and helped to draft the manuscript. All authors have read and approved the final manuscript.
